# *Animal Welfare* – the society journal for Science for Animal Welfare

**DOI:** 10.1017/awf.2026.10102

**Published:** 2026-07-28

**Authors:** Birte L. Nielsen

**Affiliations:** Science for Animal Welfare, United Kingdom

Many of you will be aware that the organisation behind *Animal Welfare* has recently changed its name to Science for Animal Welfare. This has been a long time in the planning, as the original name (Universities Federation for Animal Welfare) did not reflect or make obvious what the organisation did. By putting science into the name, Science for Animal Welfare is now showing the world what it is about: to improve the welfare of animals through a scientific understanding of their needs and how to meet them. Science for Animal Welfare does this by discovering what matters to animals, developing scientific solutions to animal welfare problems, and disseminating evidence-based information about animal welfare. This journal is at the core of this mission. *Animal Welfare* aims to be the journal of choice for the scientific community that constitutes the membership of Science for Animal Welfare and will continue to be the society’s journal.

At the recent Centenary conference for Science for Animal Welfare – held in London, UK, on 23–25 June 2026 – a symposium was organised to discuss the reproducibility of animal welfare science (see Figure 1). Here, we discussed how we can improve the stringency of the research we do. And how we can (do our best to) ensure that our findings are relevant in the real world and have a real impact on the welfare of animals. This journal has always had a compulsory *Animal Welfare Implications* section for research articles – to compel researchers to reflect on the effects of their findings for the improvement or increased awareness of animal welfare issues. Impact is closely linked to the quality of the science, and to further promote this, we have recently created another Collection for the journal of articles on Experimental Methods and External Validity. In this Collection, we include studies on the rigour of experimental methods and to what extent the protocols we use in animal welfare science have both internal and external validity; this also includes studies on replicability and reproducibility (see Figure 1).

**Figure 1.**
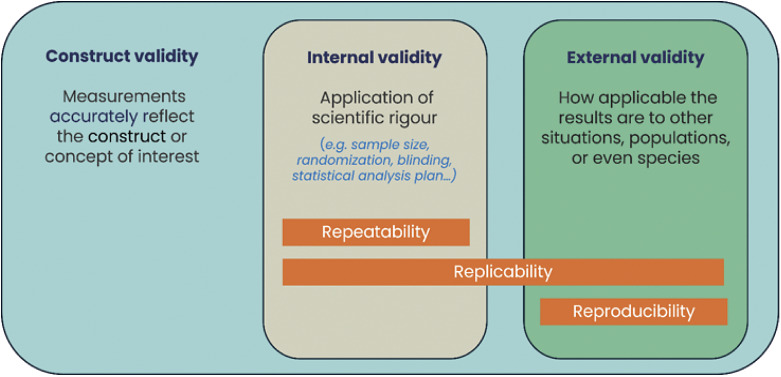
Diagram outlining the three validities of science (construct, internal, and external validity; Würbel 2017) and how the ‘other’ three Rs relate to this: *repeatability* is used for experimental repetitions within a laboratory, sometimes within the same study, whereas *replicability* is the degree to which the protocol can be replicated by other researchers in other laboratories. *Reproducibility* is then to what extent comparable results are obtained (see also Plesser 2018; iRISE 2024). Figure 1 long description.

In this Collection, we would also like to include articles that try to reproduce often-cited studies which have only been done once or only in one lab. One example of this is the used-in-many-textbooks example by Tinbergen (1939) of the stress reactions of young poultry chicks when a silhouette of a hawk or a goose was flown over them. When repeated by Schleidt (1961; reanalysed by Schleidt *et al.* 2011), the results indicated that it is the novelty of the shape rather than the short neck of the bird silhouette that caused the response observed, as also originally put forward by Lorenz (1939; a more detailed presentation of this can be found in Chapter 11 in Nielsen 2020). We therefore encourage our readers to think of other examples of such ‘done once, cited much’ research and to try and replicate these – preferably pre-registering their protocol beforehand.

We really hope that you like the new name for our society – or at least can follow the reasons for the change. The cover of the journal has been updated with the new name and logo for Science for Animal Welfare. And the journal continues to strive to be the most sought-after journal in the area of animal welfare science, both in terms of references and publications. The continued increase in our impact factor is a testament to this – brought about by the great work submitted by our authors, and the fantastic job carried out by our Section Editors and, not least, our reviewers. Thank you all for making this journal continue to grow by the quality papers your produce, thereby assisting Science for Animal Welfare in one of its missions: sharing the animal welfare evidence with anyone and everyone so it can be used to improve the lives of animals.

## Figure 1 Long description

Diagram showing three boxes titled construct, internal and external validity, the first box enclosing the other two. Three bars run horizontally, with the words repeatability, replicability and reproducibility on each, respectively. Repeatability is within the internal validity box, replicability also covers the external validity box, whereas reproducibility only covers the last box.

Navigate back to Figure 1.
